# Acute Pancreatitis Caused by Hemobilia: An Unusual Complication of Laparoscopic Cholecystectomy in a Patient With History of ROUX-EN-Y Gastric Bypass

**DOI:** 10.14309/crj.0000000000001721

**Published:** 2025-05-24

**Authors:** Adily N. Elmi, Sami Mesgun, Arthur R. Baluyut

**Affiliations:** 1 Department of Medicine, Case Western Reserve University, Cleveland, OH; 2 University Hospitals Cleveland Medical Center, Cleveland, OH; 3 Northside Gastroenterology, Indianapolis, IN

**Keywords:** Hemobilia, laparoscopic cholecystectomy, acute pancreatitis, hepatic artery pseudoaneurysm, Roux-en-Y gastric bypass

## Abstract

Acute pancreatitis as a result of hemobilia after laparoscopic cholecystectomy is a rare vascular complication with a challenging clinical diagnosis and treatment approach not eminent or available. We are reporting the fourth case of acute pancreatitis after laparoscopic cholecystectomy caused by hemobilia secondary to a right hepatic artery pseudoaneurysm. To our knowledge, this is the first such case reported in the United States.

## INTRODUCTION

Hemobilia refers to extravasated blood in the biliary tract. The most common causes of hemobilia are iatrogenic, traumatogenic, and neoplastic. Although hemobilia remains an uncommon cause of gastrointestinal bleeding, its incidence has gradually increased due to widespread hepatopancreatobiliary procedures. Hemobilia classically presents with the triad of jaundice, right upper quadrant (RUQ) pain, and upper gastrointestinal bleeding (UGIB); however, presentation often depends on the etiology. Nevertheless, diagnosing hemobilia can be clinically challenging, and the ideal treatment approach may not be immediately clear or readily accessible.^1^

## CASE REPORT

A 56-year-old man with a history of Roux-en-Y gastric bypass surgery, with bypass revision 1 year before LC, presented to the emergency department with 2 days history of sharp, squeezing epigastric and RUQ pain. He also reported 1 episode of bright red blood per rectum. Three weeks earlier, he had undergone a challenging LC due to significant gallbladder (GB) inflammation and was discharged on postoperative day 2. Physical exam revealed mild diffuse abdominal tenderness and hypoactive bowel sounds. Laboratory studies revealed a hemoglobin level of 11.4 mg/dL, leukocyte count of 15,000/mm^3^, total bilirubin of 2.5 mg/dL, aspartate aminotransferase (638 U/L), alanine aminotransferase (564 U/L), alkaline phosphatase (814 U/L), and lipase (15,904 U/L).

**Figure 1.**
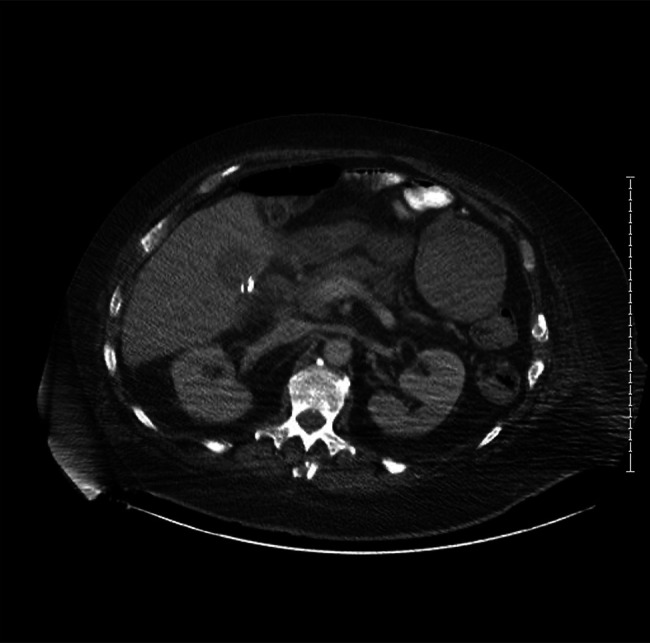
Axial computed tomography image shows a nonspecific fluid collection in the gallbladder fossa and subtle haziness anterior to the pancreatic head.

RUQ ultrasound showed a dilated common bile duct (CBD) with amorphous debris and inflammatory changes in the pancreatic head on computed tomography (CT) (Figure 1). He was diagnosed with AP based on symptoms, and elevated lipase, and he was managed conservatively. His hospitalization was complicated by a second episode of pancreatitis and ongoing hematochezia, which prompted expedited evaluation with colonoscopy and esophagogastroduodenoscopy (EGD).

**Figure 2.**
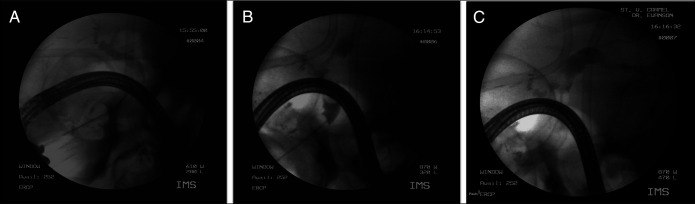
(A) Endoscopic retrograde cholangiopancreatography extraction balloon via guidewire of large blood clots. (B) Refilling of the bile duct with blood was noted after balloon extraction of large clots. (C) Biliary stent placed to maintain bile duct patency and prevent obstruction via recurrent blood clots.

**Figure 3.**
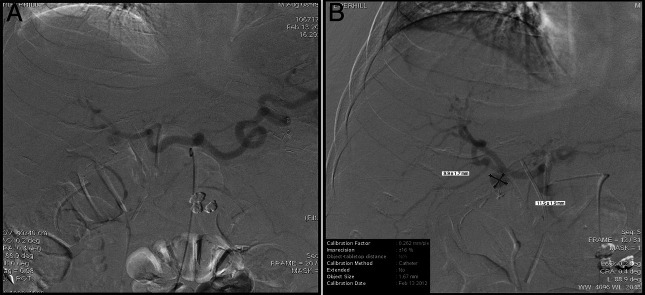
(A) Digital subtraction angiography revealing patent proximal right hepatic artery. (B) Right hepatic artery pseudoaneurysm located at the bifurcation of its 2 main branches.

**Figure 4.**
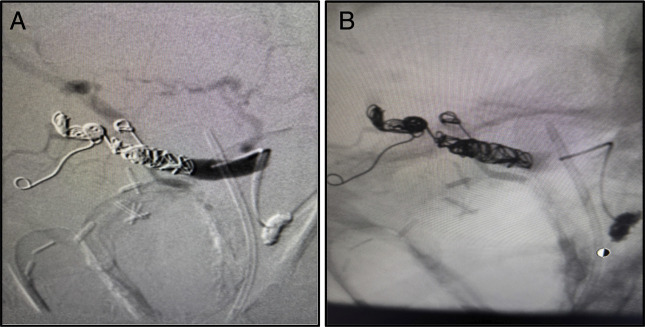
(A) Coil embolization deployed in subbranches of the posterior-inferior right hepatic artery. (B) Digital subtraction angiography confirms absence of flow through right hepatic artery and the pseudoaneurysm.

Due to his altered anatomy, a transgastric EGD was performed and hematin was evident in the gastric lumen, and duodenal bulb, no ulcers were evident. After further advancement, the ampulla came into view and with evidence of bleeding. Transgastric EGD was converted to endoscopic retrograde cholangiopancreatography (ERCP). Sphincterotomy was performed, which then revealed blood clots obstructing the CBD, and active bleeding, which was not brisk (Figure 2). After balloon dilation and sweeping the debris, a double flanged biliary stent was placed. Mesenteric angiography was performed due to the presence of hemobilia, and angiography confirmed a 1.2 cm × 1 cm right hepatic artery pseudoaneurysm (HAP) at the bifurcation of the right and left hepatic artery (Figure 3), which was successfully coil-embolized (Figure 4). The patient's hemoglobin (Hgb) dropped from 10 g/dL to 6.5 g/dL and incremented to 8.5 g/dL after transfusion of 3 units of packed red blood cells.

This decline likely reflected correction of hemoconcentration, as postembolization blood loss was minimal. The patient remained hemodynamically stable in the intensive care unit with stable Hgb, transferred to a general medicine floor, and discharged 5 days later. The biliary stent was removed 4 months later at the patient's request and partly due to delays in coordinating transgastric ERCP with general surgery. No complications were reported, and he remained asymptomatic at 6-month follow-up.

## DISCUSSION

Hemobilia refers to the presence of blood within the biliary tract, a term first coined by Sandblom^2^ in his paper titled: “Hemorrhage into the biliary tract following trauma.” Hemobilia occurs when an abnormal communication forms between the splanchnic vasculature and the biliary tree, leading to blood extravasation. Classically, hemobilia presents with Quincke's triad of jaundice, RUQ pain, and UGIB. However, this classic triad is observed in only 22% to 35% of cases, making the diagnosis challenging.^3,4^

In recent years, the increasing prevalence of hepatobiliary interventional procedures has led to a shift in the primary etiology of hemobilia. Iatrogenic causes now account for most cases, surpassing accidental trauma. Less commonly, hemobilia may result from HAP, among other vascular abnormalities.^5^

AP following after LC is rare and is usually due to pancreatic duct obstruction by retained stones. Most patients pass the stone, but those with chronic obstruction may require ERCP to clear stones from the CBD. HAP after LC is a rare but recognized complication that may present as hemobilia.^6^ Provoking factors such as mechanical or thermal injury and clip encroachments during the procedures have been implicated. In our case, injury to the vasculature was likely due to our patient being a poor surgical candidate and repeat gastric bypass surgeries.

Hemobilia causing AP has been previously reported, with most cases linked to liver biopsy, transhepatic drainage, and transhepatic cholangiography. In our case, AP was triggered by hemobilia resulting from a right HAP after LC. To our knowledge, there have been only 3 other cases reported worldwide.^7–9^ The pathogenesis involves the formation of intrabiliary clots due to hemobilia, which subsequently leads to obstruction of the pancreatic duct.

Although diagnosis can be elusive, clinicians should be highly suspicious when AP due to hemobilia manifests along with signs and symptoms of UGIB. Upper endoscopy with direct visualization of blood or clot from the biliary tract essentially confirms the diagnosis, and ERCP can be used to further visualize the biliary tree and may offer therapeutic options. CT angiography is the diagnostic modality of choice for hemobilia and has replaced catheter arteriography for diagnosis of pseudoaneurysms.^10^ Our case utilized celiac arteriogram with delayed phase to diagnose HAP as CT angiography was not an available imaging modality.

Management should focus on achieving hemostasis and maintaining bile flow. Transcatheter arterial embolization (TAE) is the initial treatment of choice as it permits distal as well as proximal control of the hepatic artery. TAE should be avoided in patients with liver allografts, cirrhosis, and portal vein thrombosis given such patients have compromised collateral blood flow from the portal vein, as a result of which TAE can lead to ischemic liver injury.^11^ If TAE fails, the next step in management is surgery. Complications due to laparoscopic and laparotomic surgeries performed near the cystic and right hepatic artery have been implicated as a cause of hemobilia. Often these surgeries have been reported to cause hemobilia through the formation of HAP.^12^ More specific to our case, vascular injuries during LC may occur in 0.2%–0.5% of cases, and these injuries are also reported to cause hemobilia with pseudoaneurysmatic dilatation.^13^

Hemobilia arising from HAP after LC and causing AP is extremely rare and diagnosis can be challenging as evidenced in our patient. Our case further demonstrates how timely diagnosis can be delayed in patients with altered anatomy such as gastric bypass surgery. We, therefore, highlight a critical complication of LC, emphasizing that timely recognition and intervention are essential for minimizing morbidity and mortality.

## DISCLOSURES

Author contributions: AN Elmi: Literature review, wrote and revised the manuscript and provided final approval of the manuscript. S.Mesgun: Revision of the manuscript and provided final approval of the manuscript. AR Baluyut: The acquisition of the case report, obtained informed consent, provided radiological images, critical revision of the article for important intellectual content.

Financial disclosure: None to report.

Previous presentation: This case was presented at the American College of Gastroenterology Annual Scientific Meeting; October 25–30 2024; Philadelphia, PA.

Informed consent was obtained for this case report.
